# Zosteriform skin metastasis caused by retrograde lymphatic migration of metastatic squamous cell lung carcinoma

**DOI:** 10.1186/s12890-021-01414-9

**Published:** 2021-01-26

**Authors:** Yohei Maki, Yoshifumi Kimizuka, Koji Murakami, Kimiya Sato, Hisashi Sasaki, Takayuki Yamamoto, Chie Watanabe, Tomoya Sano, Jun Miyata, Yuji Fujikura, Akihiko Kawana

**Affiliations:** 1 Division of Infectious Diseases and Respiratory Medicine, Department of Internal Medicine, National Defense Medical College, 3-2, Namiki, Tokorozawa, Saitama 359-8513 Japan; 2 Department of Radiology, Juntendo University School of Medicine, 3-1-3, Hongo, Bunkyo-ku, Tokyo 113-8421 Japan; 3 Department of Basic Pathology, National Defense Medical College, 3-2, Namiki, Tokorozawa, Saitama 359-8513 Japan

**Keywords:** Zosteriform skin metastasis, Lung cancer, Cancerous lymphangiopathy, Autopsy, ^18^F-fluorodeoxyglucose-positron emission tomography, Computed tomography

## Abstract

**Background:**

Zosteriform skin metastasis (ZSM) is rare, and its etiology is not well understood. ZSM is possibly derived from the retrograde movement of cancer cells through the lymphatic vessels during disease development. However, it has been difficult to demonstrate it, as no specific findings have been observed.

**Case presentation:**

A 68-year-old man presented to our department with neck lymphadenopathy. After detailed examinations, squamous cell lung carcinoma (cT2aN3M1c) was diagnosed. Although cisplatin combined with gemcitabine was administered, his cancerous lymphangiopathy was exacerbated, and ZSM was observed on his right chest. Pembrolizumab was initiated as a second-line chemotherapy; however, the patient died 7 months after the initial presentation. In this case, fluorodeoxyglucose-positron emission tomography indicated the presence of skin metastasis and cancerous lymphangiopathy. Similarly, after performing an autopsy, tumor-cell filled lymph ducts were observed in the right subclavian and the cutaneous lymphatic vessel from the right hilar lymph nodes.

**Conclusions:**

To the best of our knowledge, this is the first study to demonstrate that the localization of ZSM in the cutaneous lymphatics was caused by the retrograde movement of cancer cells through the lymphatic vessels, using radiographical and pathological analysis. In addition, fluorodeoxyglucose-positron emission tomography may help predict skin metastasis induced by cancerous lymphangiopathy.

## Background

Zosteriform skin metastasis (ZSM) develops on the skin in regions similar to those where herpes zoster manifests. However, ZSM is rare, and reports of ZSM development from primary lung cancer lesions are limited [1–5]. Although its etiology is unknown, two hypotheses may explain ZSM development. One hypothesis involves a Koebner-like reaction at the site of a past varicella-zoster virus (VZV) infection [6], while another involves the retrograde movement of cancer cells through lymphatic or vascular vessels [7]. Using ^18^F-fluorodeoxyglucose positron emission tomography/computed tomography (FDG-PET/CT) and an autopsy, we report the first case of ZSM development linked to the retrograde mechanism, as a result of cancerous lymphangiopathy.

## Case presentation

**Fig. 1:**
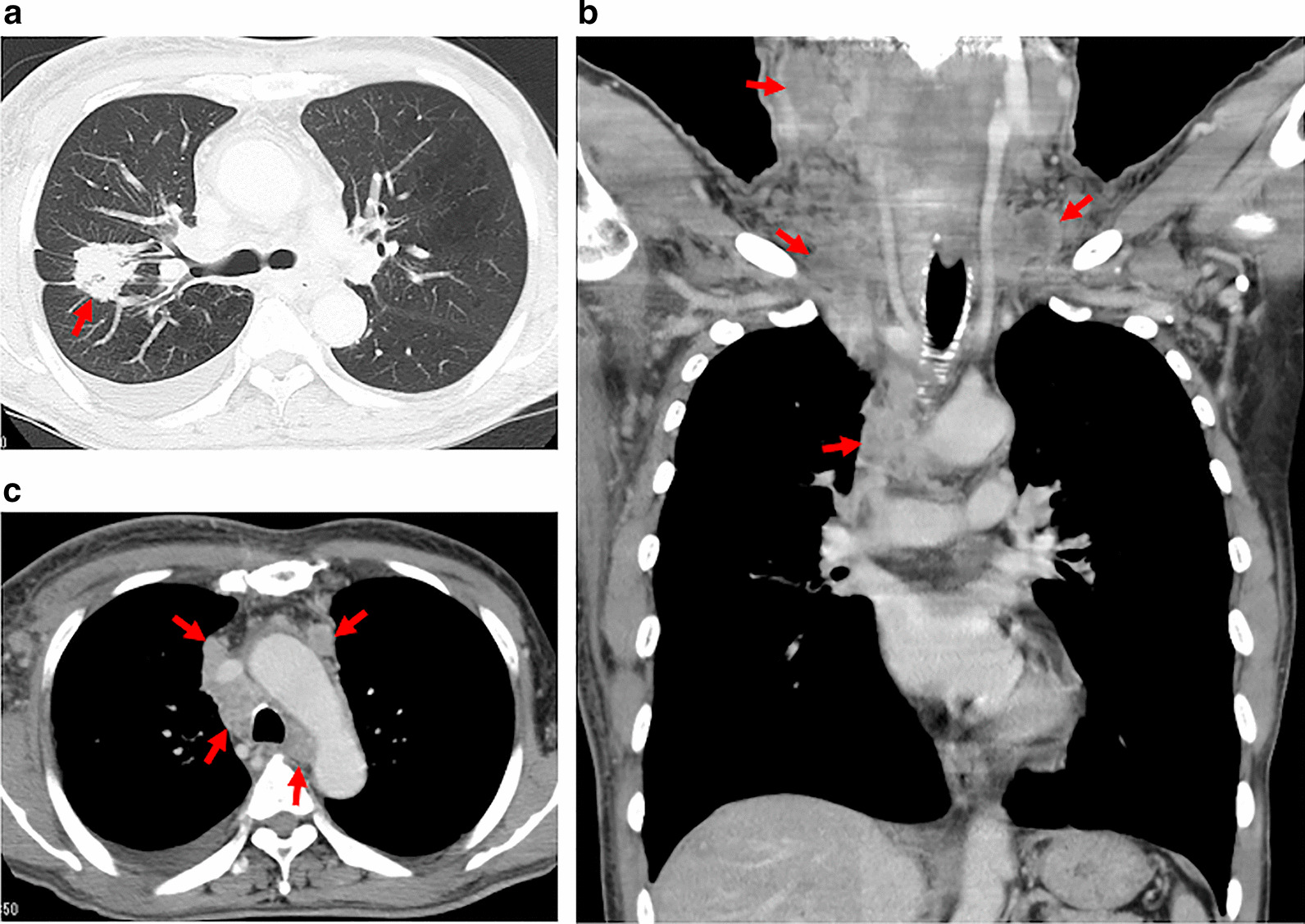
Chest computed tomography findings. **a** A tumor in the right upper lobe with hypertrophy of the bronchovascular bundles (arrow). **b** Lymphadenopathy of the hilar and mediastinal lymph nodes (arrows). **c** Lymphadenopathy of the mediastinal, supraclavicular, and cervical lymph nodes right dominantly (arrows)

**Fig. 2:**
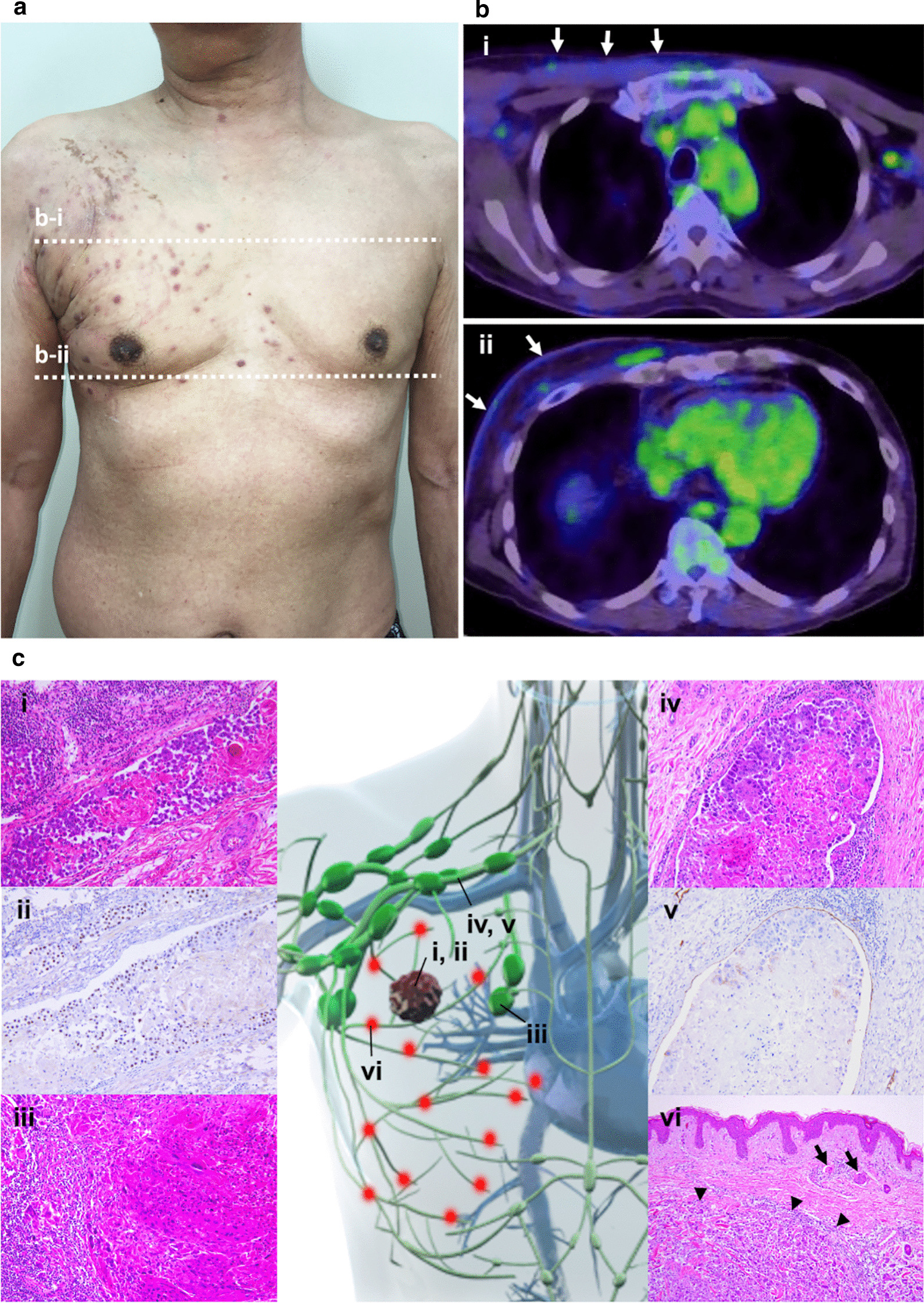
Images of the lesion. **a** Papules localized to the right anterior and lateral chest wall. **b** FDG-positron emission tomography/computed tomography findings at the level of the axilla (**b-i**) and the nipple (**b-ii**). Mild uptake of ^18^F- fluorodeoxyglucose is observed in the subcutaneous tissue (arrows). **c** Schema with histological findings. **i**, **ii**: A squamous cell carcinoma showing marked lymph vessel permeation (**i**: hematoxylin and eosin; **ii**: p40 immunostaining, original magnification × 200); **iii**: A metastatic squamous cell carcinoma with degeneration in the right pulmonary hilar lymph node (hematoxylin and eosin stain, original magnification × 200); **iv**, **v**: Remarkable lymphatic vessel invasion in the subclavian lesion (**iv**: hematoxylin and eosin; **v**: D2-40 immunostaining, original magnification × 200); **vi**: The metastatic skin nodule (arrowheads) with lymph vessel permeation (arrows) (hematoxylin and eosin stain, original magnification × 100)

A 68-year-old man presented to our department with neck lymphadenopathy. His medical history revealed that he had undergone endoscopic resection of a colon polyp 1 year previously, and he used to smoked two packs of cigarettes per day for 48 years. His vital signs were stable, bur his blood pressure and pulse rate were elevated (148/91 mmHg [normal values: ≤ 120/80 mmHg] and 111 bpm [normal values: 60–100 bpm], respectively). Physical examination revealed edema from the neck to the right shoulder and swelling of the right postauricular, cervical, and axillary lymph nodes. Chest CT revealed a tumor in the right upper lobe with hypertrophy of the bronchovascular bundles (Fig. 1a). Lymphadenopathy was apparent at the hilar, mediastinal, supraclavicular, axillary, and cervical lymph nodes, predominantly on the right (Fig. 1b, c). The superior vena cava was slightly compressed but patent. Right cervical lymph node biopsy and cytology of the sputum revealed lung squamous cell carcinoma (cT2aN3M1c; programmed death ligand 1 tumor proportion score, 10%). Although cisplatin plus gemcitabine treatment was performed in six cycles and reduced the size of primary lesion, the lymphedema progressed, and papules appeared in the anterior and lateral chest wall (Fig. 2a). FDG-PET/CT revealed scattered FDG uptake in the skin around the papules (Fig. 2b, Additional file 1: Fig. S1). Skin biopsy indicated ZSM, which was judged as progressive disease. Pembrolizumab was administered, as a second-line chemotherapy, for two cycles; however, cancerous lymphangiopathy worsened and the patient died 7 months after the initial presentation.

An autopsy, and post-mortem biopsy of the skin and cervical lymph nodes indicated metastatic primary squamous cell lung carcinoma. In addition to the bilateral hilar lymph node metastasis, which was significantly larger on the right side, the lymphatic vessels near the subclavian vein and the lymphatic vessels inside the dermis near the papules were filled with tumor cells (Fig. 2c).

## Discussion and conclusions

We present the case of a patient with ZSM developed from lung cancer, as a result of retrograde metastasis of cancer cells through the lymphatic vessels. The retrograde mechanism of ZSM development has previously been supported by qualitative lymphoscintigraphy analysis [1]. To our knowledge, this is the first report that used pathological data from autopsy report and radiological FDG-PET/CT data to confirm the development of ZSM via the lymphatic retrograde migration of cancer cells.

The pathogenesis of ZSM is hypothesized to occur as follows: (1) A Koebner-like reaction at the site of past VZV infection; and (2) retrograde migration via the lymphatics or blood vessels [6, 7]. In our case, the lack of past medical history of skin disease and the continuous tumor regurgitation from the hilar lymph nodes to the dermal lymph ducts via the ipsilateral mediastinal, subclavicle, and axillary lymph nodes, as confirmed on autopsy, led us to conclude that ZSM developed through the latter mechanism (Fig. 2c).

In this case, we observed ZSM using FDG-PET/CT. It is generally difficult to confirm whether an FDG-PET/CT finding is a cancerous lymphatic regurgitation or simple lymphedema [8]. In cases of lymphedema, FDG is taken up by tissues slightly but uniformly [8]. In contrast, this case showed distinctive FDG uptake patterns that matched the locations of the skin metastasis. Based on these observations and the histology of autopsy specimens, we speculated that the FDG-PET/CT features may help diagnose lymphatic retrograde skin metastasis.

However, there were some limitations that should be acknowledged. First, pathological demonstration of metastasis to the right axillary lymph nodes, which is the confluence of lymph vessels in the chest wall, was not performed because these samples were not collected during autopsy. However, ^18^F-FDG uptake was similarly observed in the skin and the subclavicle and axillary lymph nodes. Τherefore, we hypothesized that there were also metastatic sites in the axillary lymph nodes. Second, we could not completely exclude the possibility that the Koebner-like reaction mechanism caused the ZSM formation. In some cases, the detection of viral DNA in the skin lesions of ZSM was useful for the demonstration of the mechanism, but in many cases, this method proved unsuccessful, thus, suggesting a more complicated pathologic mechanism of Koebner-like reaction of ZSM [6]. Given that the patient in this case had no history of zoster, we believe that it is unlikely that VZV infection contributed to the ZSM development.

In conclusion, using FDG-PET/CT and an autopsy, we demonstrated that ZSM developed through the retrograde migration of cancer cells to the right anterior thoracic lymphatics, via the hilar, mediastinal, subclavian, and axillary lymph nodes/vessels in a patient with lung cancer. Although pathological proof was essential, FDG-PET/CT may be useful to evaluate metastasis progression and predict complications, including cancerous lymphangiopathy and skin metastasis.

## Supplementary information

**Additional file 1: Fig. S1.** ^18^F-Fluorodeoxyglucose (FDG)-positron emission tomography (PET)/computed tomography image with a narrowed window width on PET. FDG uptake on the skin and the subcutaneous tissue around the papules at the level of the axilla (**i**) and the nipple (**ii**) (arrows). In these images, the window width on PET appears to be narrow to highlight the uptake in the subcutaneous tissue.

## Data Availability

The datasets used and analyzed during the current study are available from the corresponding author on reasonable request.

## Abbreviations

CT: Computed tomography
FDG: ^18^F-Fluorodeoxyglucose
PET: Positron emission tomography
VZV: Varicella-zoster virus
ZSM: Zosteriform skin metastasis
