# Supplementary figures and images for: Zosteriform skin metastasis caused by retrograde lymphatic migration of metastatic squamous cell lung carcinoma
Source: BMC Pulm Med. 2021 Jan 26;21:41. doi: 10.1186/s12890-021-01414-9 (PMC7836447; doi:10.1186/s12890-021-01414-9)

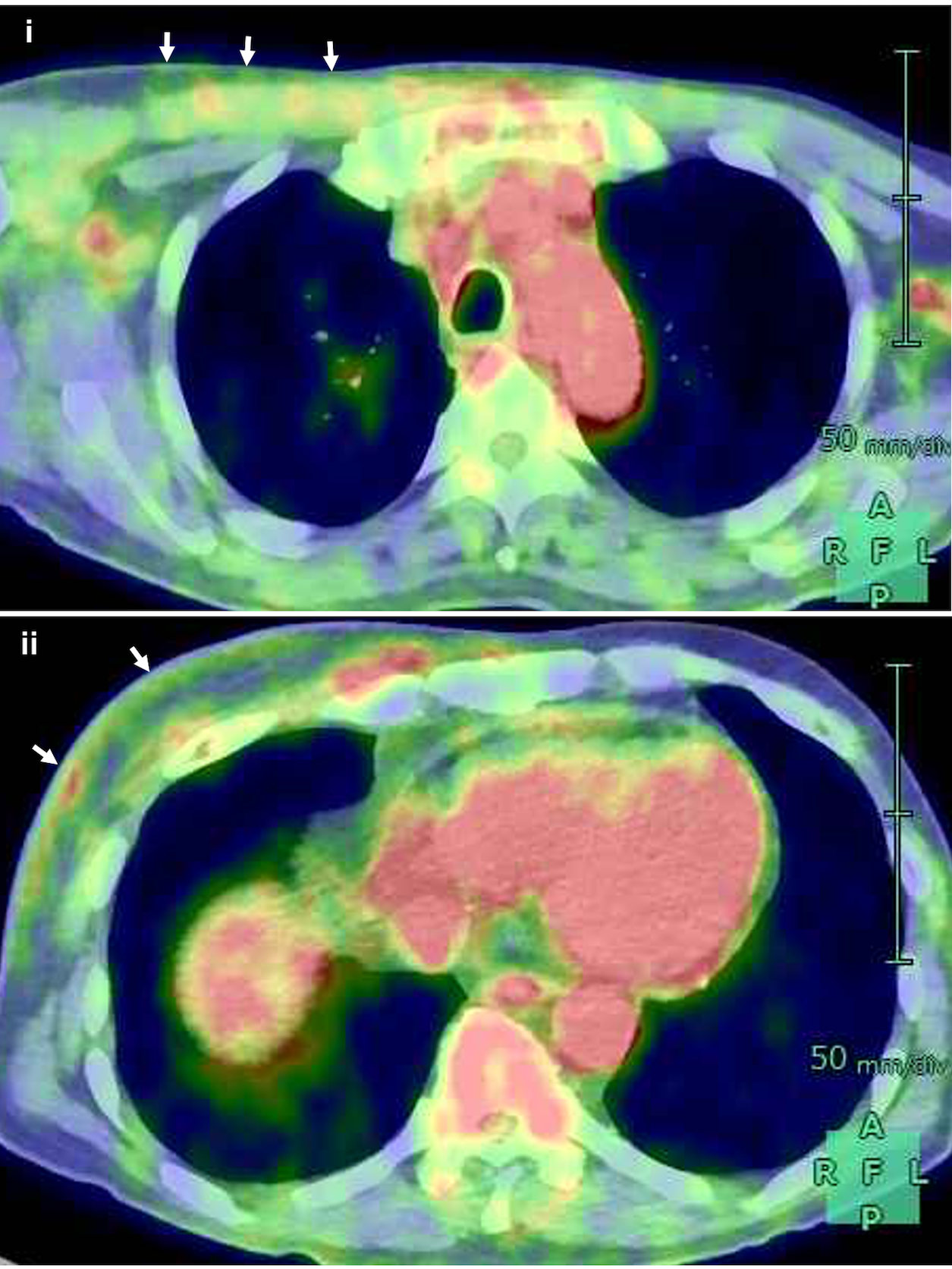

Supplement: Supplementary file 1 — Additional file 1: Fig. S1. 18F-Fluorodeoxyglucose (FDG)-positron emission tomography (PET)/computed tomography image with a narrowed window width on PET. FDG uptake on the skin and the subcutaneous tissue around the papules at the level of the axilla (i) and the nipple (ii) (arrows). In these images, the window width on PET appears to be narrow to highlight the uptake in the subcutaneous tissue. [file 12890_2021_1414_MOESM1_ESM.tiff]
